# Conspiracy Beliefs and Monothematic Delusions: A Case for De-pathologizing

**DOI:** 10.1007/s10670-024-00881-w

**Published:** 2024-11-25

**Authors:** Anna Ichino, Ema Sullivan-Bissett

**Affiliations:** 1 University of Milan, Milan, Italy; 2 University of Birmingham, Birmingham, UK

## Abstract

Monothematic delusions and beliefs in conspiracy theories share some important features: they both typically have bizarre contents and are resistant to counterevidence. Yet conspiracy beliefs are generally taken to be a normal range phenomenon, whilst monothematic delusions are considered to involve doxastic pathology. In this paper, we argue that this difference in conceptualization is not warranted, and that, if we’re right, the correct response is to de-pathologize monothematic delusions.

We identify three reasons which could justify taking monothematic delusions to be pathological beliefs whilst not treating conspiracy beliefs in such terms. First, we consider what have been identified as initial provoking conditions in monothematic delusions (anomalous experience) and conspiracy beliefs (epistemic mistrust). Second, we consider the role of cognitive biases or deficits in these phenomena. Third, we consider the different roles played by testimony and one’s social environment in the formation and maintenance of monothematic delusions and conspiracy beliefs. We argue that there are no grounds from any of these quarters for the different approaches taken with respect to the question of doxastic pathology. Our primary conclusion then is that monothematic delusions and conspiracy beliefs should be treated the same in this respect. Our secondary conclusion is that the correct way to do so is to de-pathologize monothematic delusions.

## Preliminaries

Conspiratorial ideation is widespread, and the idea that the beliefs resulting from it are manifestations of psychopathology has been recurrent in the research on this phenomenon at least since Richard Hofstadter’s seminal essay ‘The Paranoid Style in American Politics’ (1964). Language suggestive of a similar view remains present in the public debate where conspiracy theorists are often described as ‘delusional’.^1^ However, a lot of recent research has resisted the idea that these beliefs are pathological and most psychologists seek to explain the formation of these beliefs by identifying normal range cognitive contributions. So, for instance, Joseph Pierre describes conspiratorial ideation as ‘essentially a normal phenomenon’ (2020: 618), and goes on to argue that the kinds of biases and intellectual styles associated with conspiracy beliefs are widespread. Rob Brotherton claims that ‘conspiracy theories are not exclusively a feature of the fringe (…) but thrive in the mainstream, too’ (2015: 104). Scott Radniz and Patrick Underwood argue that ‘rather than a marginal phenomenon, scholars should study conspiracy theories as a natural byproduct of political reasoning under uncertainty’ (2015: 126).^2^ As Joseph Uscinski points out in his comprehensive historical overview of conspiracy research: ‘the *pathologizing paradigm* that was once more popular has now been abandoned, leaving room for the increasing recognition that conspiracy theories are a mainstream phenomenon’ (Uscinski 2018: 37).

This of course is compatible with conspiracy theories being understood as a negative and problematic phenomenon. Some authors build this negativity into the definition of conspiracy theories, aligning their approach with everyday language and folk understanding (Napolitano and Reuter 2021). On these pejorative approaches, ‘conspiracy theory’ is not simply a theory that refers to a conspiracy, but is a theory that refers to a conspiracy *while also* suffering from some (typically epistemic) problems, such as evidential self-insulation (see e.g. Cassam, 2019; Napolitano, 2021) or being grounded in a ‘conspiracy mindset’ (Ichino 2024). Other authors defend a neutral, minimal definition, according to which a conspiracy theory is simply a theory that refers to a conspiracy as a salient cause of the phenomena it seeks to explain (see e.g., Dentith, 2014; Duetz, 2022).^3^ Here we assume a pejorative definition, which is shared, more or less explicitly, in much of the empirical literature that we discuss. Nevertheless, our arguments do not depend on that definition, since neither advocates of pejorative definitions nor advocates of neutral definitions take beliefs in conspiracy theories to be pathological.

Let us turn now to delusion, starting with the characterisation given in the most recent edition of the Diagnostic and Statistical Manual of Mental Disorders. There they are characterised as:

fixed beliefs that are not amenable to change in light of conflicting evidence. Their content may include a variety of themes (e.g. persecutory, referential, somatic, religious, grandiose) […] Delusions are deemed bizarre if they are clearly implausible and not understandable to same-culture peers and do not derive from ordinary life experiences […] (DSM-5 2013: 87)

This characterisation of delusion is contentious to say the least (see Sullivan-Bissett, 2024a: 3–6 for discussion), so let us also note some examples to get the phenomenon in sight. In Capgras delusion someone believes that *a familiar person (i.e. a loved one) has been replaced by a near-identical looking imposter*, in Cotard delusion someone believes that *they are dead* or *have ceased existing*, in anosognosia someone believes that *they do not have a physical impairment* (when they do). We will focus on *monothematic* delusions (the examples just noted often present monothematically), which concern a single theme, and often occur in the absence of a more general delusional belief system. We do so in line with the theories of delusion formation we discuss later.^4^ From this point, ‘delusion’ should be taken to refer to *monothematic* delusion.

Few researchers would resist the idea that delusions are pathological beliefs. Many recent discussions of the topic begin by asserting the pathological status of delusion as a datum, and then proceed to give accounts of what makes it so. To give some examples: Lisa Bortolotti begins her Stanford Encyclopaedia entry on delusion by saying: ‘let’s assume that delusions are pathological beliefs’ (Bortolotti, 2018, cf. her 2022); Valentina Petrolini opens her paper by saying that Bortolotti’s analysis of delusion ‘leaves an important question unanswered: What makes delusions pathological?’ (2017: 502); Kengo Miyazono opens his paper with the claim that: ‘delusional beliefs are typically pathological’ (2015: 561); and Eisuke Sakakibara similarly notes that: ‘[d]elusions are currently thought of as pathological beliefs’ (2016: 147). Think also of Max Coltheart and Martin Davies’s (1991) collection of essays on delusion entitled *Pathologies of Belief*.

We thus take this as our starting point: delusions are often taken to be pathological beliefs, whilst conspiracy beliefs are not. Our primary aim in what follows is to argue that the differences between conspiracy beliefs and delusions do not justify this divergence. That’s not because the differences fail to get conspiracy beliefs off the hook of pathology, but rather because they fail to get delusions on that hook. Thus, our secondary aim is to argue that we should take neither to involve doxastic pathology. The case for our claims is conditional, that is, if certain mainstream approaches to these phenomena are broadly right, then there are no grounds for treating conspiracy beliefs and delusional beliefs as different with respect to doxastic pathology.

Of course, our arguments are not intended to rule out the possibility that a conspiracy belief or a delusion *could* arise from doxastic pathology. However, that possibility ought not be mistaken as providing insight into the nature of these beliefs as they arise in general. And, in general, we say, (1) the differences between the two kinds of belief does not justify the divergence in approach to pathological status, and (2) the evidence suggests that neither involve doxastic pathology.

In Sect. 2 we give an overview of some accounts of *pathology in belief,* and pin down a version of the popular etiological account we will take forward. The rest of the paper thus explores the etiological differences between conspiracy beliefs and delusions.

In Sect. 3 we consider *provoking conditions*, comparing the etiological role of what has been identified as the first factor in delusional beliefs (anomalous experience), and asking what might play an analogous role in the case of beliefs in conspiracy theories. We identify epistemic mistrust as a candidate, and we argue that neither anomalous experience nor epistemic mistrust can be the grounds for doxastic pathology.

In Sect. 4 we turn to the role of *cognitive biases and deficits* hypothesized to be key to the formation of conspiracy beliefs and delusions. In the case of conspiracy beliefs, the psychological literature has sought to identify a variety of normal range cognitive contributions, whilst the orthodoxy in delusion research has been to seek out clinically significant reasoning abnormalities. We argue that this difference in approach is unwarranted.

In Sect. 5 we discuss the idea that beliefs in conspiracy theories have considerable *social currency* and are maintained in part by dynamics of group identity and belonging, whilst delusions have themes rather removed from society and culture and are usually not shared among same culture peers. We argue that the differences here may be less sharp than they seem, and that, in any case, they do not justify treating conspiracy beliefs and delusional beliefs as different with respect to doxastic pathology.

Two final preliminaries before we proceed. First, in what follows we assume doxasticism for both conspiracy attitudes and delusions. The debates we discuss regarding anomalous experience, epistemic mistrust, cognitive bias, social currency, and so on, also proceed, by and large, by taking these attitudes to be beliefs, and so we are in line with assumptions framing these debates. Our overarching aim is methodological: we argue that the difference in approach with respect to doxastic pathology in research on conspiracy beliefs and delusions is not warranted. Given that doxasticism represents the current orthodoxy in both cases, we align our arguments with that background assumption.^5^

Second, although we talk of conspiracy beliefs and delusions as kinds of belief, our arguments do not adjudicate on whether such beliefs constitute *natural* kinds. Our view concerning whether the differences between conspiracy beliefs and delusions justify taking (only) the latter to be pathological is consistent with natural kindhood for either kind of belief, but does not require it. We do generalize over conspiracy beliefs and monothematic delusions, but in doing so we are not thus committed to natural kindhood in either case. Generalisations would be very expensive if they were only legitimate for natural kinds. We follow the approach of other researchers in these areas in taking the beliefs in each group to be similar enough to warrant generalising over them. And, as we noted above, our view is open to the idea that *some* conspiracy beliefs and delusions do in fact arise from doxastic pathology – nothing we say here rules that out. Rather, we argue that our starting point in understanding the nature of such beliefs ought not reside in a framework of doxastic pathology.^6^

## Pathology and Belief

Our interest in this paper is *doxastic* pathology, and so we note at the outset that our arguments for parity of treatment with respect to this are consistent with the claim that pathology of a different kind may nevertheless be present. It might be thought that the argument that conspiracy beliefs and delusions are not pathological *beliefs*, whilst granting that they may involve a different kind of pathology, may be right in principle, but is mere philosophical pedantry. However, locating precisely where the pathology lies *is* important. Doing so helps us better understand the nature of these beliefs, in particular, it might speak to whether they can be treated as continuous with other irrational beliefs (some authors take doxastic pathology to be inconsistent with continuity, see e.g. Petrolini 2024). Being precise about where pathology lies might also be relevant to therapeutic intervention: in order for it to be successful, we must know where to focus our efforts. Determining whether the pathology is a pathology *of belief* or *of some different mechanism* is therefore something we should care about, and not only from a philosophical point of view.^7^

With that said, let us turn to surveying some characterisations of pathological belief, before identifying the one that will form the backdrop of the discussion to follow.

### Epistemic Criterion

One view is that pathological beliefs are those which (to a given extent) flout epistemic norms. For example, if a belief is formed or maintained on insufficient evidence, or if the evidence produced is not plausibly evidence for the believed content, or if it is maintained in the face of blatant counterevidence, then the belief is pathological.

Now, there is some presumed guilt when it comes to how we think of delusions and their relationship to evidence. Characterisations of delusions in the most recent two versions of the DSM have it that delusion is a false belief ‘firmly sustained despite what almost everyone else believes and despite what constitutes incontrovertible and obvious proof or evidence to the contrary’ (DSM-IV 2000: 765), and that a delusion is a belief held ‘despite clear or reasonable contradictory evidence regarding its veracity’ (DSM-5 2013: 87). If that is right, pathology understood as arising from a belief’s relationship to evidence would capture delusions by (diagnostic) definition.

However, it is far from clear that delusions can be distinguished from other beliefs on the basis of their relationship to evidence. In the first place, some have suggested that delusions are not transgressors in this respect after all. Many delusions are preceded by highly anomalous experiences, which might be considered a source of evidence. It has been argued that the evidential import of such experiences ought to be defeated by other considerations, most obviously, one’s background beliefs (see e.g. Coltheart and Davies 2021: 222), or testimony from others. However, it is not, in fact, obvious how one should weigh one’s experience against background beliefs (see discussion in Noordhof & Sullivan-Bissett, 2021: 10298–10300), and in some cases, taking into account the person’s full epistemic position, their delusional belief is not unreasonable (see e.g. Bayne on Capgras: 2017: 84).^8^ And, even if it appears that delusions are poorly related to evidence, we might nevertheless say that subjects with delusions retain the capacity to respond to evidence against their delusions, but that that capacity is *masked* by motivational factors and anomalous experiences functioning as reoccurring evidence for the delusion (Flores, 2021).

Second, even if delusions do massively transgress against the dictates of epistemic norms with respect to evidence, they are far from the only guilty party. Other examples of epistemically faulty beliefs might include prejudiced beliefs (Bortolotti, 2009: 124), self-enhancing beliefs (Bortolotti, Gunn, and Sullivan-Bissett 2017: 44–5), ideological beliefs (Haidt, 2013), self-deceptive beliefs (Van Leeuwen 2007: 422), and so on. A characterisation of pathological belief in terms of evidence resistance risks pathologizing too many kinds of belief.

Most importantly, the judgement that delusions are pathological and conspiracy beliefs are not would be difficult to justify using this criterion. Ian Gold and Joel Gold, in reflecting on the DSM-5’s characterisation of delusions as ‘fixed beliefs that are not amenable to change in light of conflicting evidence’ note that this ‘is no less true of a wide range of other beliefs […] conspiracy theories provide a conspicuous example’ (Gold and Gold 2024: 534). Bortolotti has it that ‘[c]onspiracy beliefs are *as resistant* to counterevidence as clinical delusion’ (2023: 64, our emphasis). Indeed, conspiracy theorists’ hasty dismissal of counterevidence has been described as ‘arguably the most important feature of conspiracy theories’ (McKenna, 2017: 57; see also Sunstein & Vermuele, 2009). And some authors, as we have seen, take this to be one of their defining features.^9^

Fans of pathologizing by appeal to epistemic features might argue that the criterion which seems to us to overreach is in fact capturing exactly the right beliefs, and that there are simply many more instances of pathological belief than is ordinarily thought. If that were right, we would have already reached our primary conclusion that the difference in judgements of pathology with respect to delusions and conspiracy beliefs is not warranted. We turn then to a different criterion of pathology.

### Bizarreness Criterion

Another approach locates the pathology of a given belief in the strangeness of its content. A belief is pathological if it meets some level of bizarreness of content. We reject this way of pathologizing belief for two reasons. First, we take it that bizarre contents can only *indicate* pathology, rather than be constitutive of it. And second, even if one thought that a belief’s bizarreness could be constitutive of its pathology, the bizarreness criterion overreaches. For example, many paranormal beliefs (e.g. beliefs in telekinesis) would presumably meet any threshold on bizarreness set for pathology (see discussion in Noordhof and Sullivan-Bissett 2023: 91), and of course our own case – that of conspiracy beliefs – will meet such a threshold too (if the belief that *Hillary Clinton and other democrats are a cabal of Satan-worshipping cannibalistic paedophiles* isn’t bizarre then we’re not sure what is!). Of course, just like with the epistemic criterion, those in favour of pathologizing by content may argue that their criterion is in fact extensionally adequate – there are simply many more pathological beliefs than we might have thought. As before, conceding this would simply get us rather quickly to our primary conclusion, namely, that different treatments of delusions and conspiracy beliefs with respect to doxastic pathology are not warranted.

A nearby idea might be put in terms of *ununderstandability,* that is, even accepting that some conspiracy beliefs and some delusions have bizarre contents, only the latter have contents which escape *understanding.* The Cotard delusion when avowed by a living breathing person might be taken to be self-defeating, the delusion that *there’s a nuclear reactor in one’s body* strikes very strong notes of physical impossibility. Perhaps the ununderstandability of (some) delusions justifies treating delusions (and not conspiracy beliefs) as pathological. We note two things in response. First, just as with bizarreness, that a belief is ununderstandable can at best be *indicative* of pathology, not *constitutive* of it. Second, as Bortolotti points out, ununderstandability may arise from the fact that we are not acquainted with the anomalous experiences that often prompt delusions (2023: 41–2), and more broadly our incredulity in the face of the expression of some delusions might arise from mere *unfamiliarity* of such contents (Bortolotti, 2023: 49). Let us turn then to the etiological criterion, which is the one we will take forward.

### Etiological Criterion

In its broadest terms, the etiological criterion has it that whether or not a belief is *pathological* depends on the mechanisms that produce it. It is natural to start from Jerome Wakefield’s (1992a) account of disorder according to which disorders are *harmful malfunctions.* Miyazono adopts this approach as a way of pathologizing belief. On his view, delusions are pathological beliefs because they involve harmful doxastic malfunctions. Sakakibara takes a similar approach, although he drops the harm component and only retains the malfunction one: ‘pathological […] beliefs are distinguished from non-pathological ones by considering whether their existence is best explained by assuming some underlying dysfunctions’ (2016: 147). Here we follow Sakakibara in dropping the harm requirement, and we strengthen the relevant notion of malfunction.

We drop the harm component for the same reason that we did not adopt the previously mentioned accounts of pathology: including it would get us straightforwardly to our primary conclusion that conspiracy beliefs and delusions ought not to be understood as different with respect to their pathological status. That’s because both kinds of belief have instances that are harmful, but also instances that are not. Although many delusions impair functioning and harm their subjects, not all do (Bortolotti et al. 2017: 45–8). For example, some delusions enhance the subject’s sense that their life is meaningful (see e.g. Ritunnano and Bortolotti 2022). In addition, the adoption of a delusion can relieve anxiety, resume learning, help the subject make sense of the world, and even deliver significant epistemic benefits not otherwise available^10^ (Bortolotti, 2020: pp. 74–89, see also Ch. 5 on motivated delusions and Bortolotti, 2023, Ch. 6 on delusions and harm). None of this is to say that delusions do this systematically or by design, it is only to say that, as a matter of empirical observation, not all delusions are harmful. On the other hand, although many conspiracy beliefs are not harmful to their subjects, some are. Karen Douglas (2021), for instance, provides evidence of both societal harm and harm concerning the subjective wellbeing of the conspiracist subject (see also Douglas et al., 2019; Jolley & Paterson, 2020). Thus, if we adopted the *harmful* malfunction version of the etiological criterion, both delusions and conspiracy beliefs would have instances which met the harm condition, and instances which didn’t. We would already be closer to our primary conclusion that different conceptualizations of these beliefs with respect to pathology is unwarranted.

Let us turn now to our strengthening of the notion of *malfunction*. In our view, Wakefield’s malfunction is too cheap for the theoretical purpose of delineating pathology. On one way of understanding doxastic malfunction, it is an everyday occurrence – examples might include cases of the kind of doxastic mistakes to which we all fall prey. These might include failing to update background beliefs, being forgetful, miscounting, misusing rules of inference, and so on. Malfunction so understood is presumably not enough to warrant verdicts of doxastic pathology given how profoundly that would overreach. Pathological beliefs would be an everyday phenomenon, and that label would thus capture many ordinary beliefs.

In light of this, let us draw a distinction between *everyday* and *abnormal* malfunction. Crucially, strengthening the relevant notion of malfunction is not to stack the deck in the discussion that follows. We will see that a natural way of pathologizing delusion and not conspiracy beliefs takes place in a conversation about normal range intellectual styles (which may well involve everyday malfunctions) versus abnormalities in mechanisms of belief formation and evaluation. The notion of *normal range* allows us to separate those malfunctions that are everyday (like errors in applying inference, failure to update background beliefs, and so on), from those malfunctions which might be relevant for judgements of doxastic pathology.

Let us identify two ways of understanding *abnormality*: functional and statistical. *Functional* normality picks out the property of being within the range of belief formation and evaluation styles which evolutionary selection has not distinguished, and functional abnormality the opposite. *Statistical normality* picks out the property of occurring in ordinary belief formation or evaluation, and statistical abnormality the opposite. Return now to our cases of everyday malfunction. Misusing rules of inferences or temporary forgetfulness may be statistically normal, but they may also be examples of functional abnormalities. But we have noted already that talk of malfunction is intended to be more substantial than this. And so, in what follows, we understand *abnormal malfunction* as picking out a functional abnormality against a statistical assumption^11^ (that is, functional abnormalities which are also statistical abnormalities are the ones constitutive of the category *abnormal malfunction*).

Wakefield himself hints at this distinction when he says:

Some cognitive mechanisms have the function of providing a person with the capacity for a degree of rationality as expressed in deductive, inductive, and means-end reasoning (I am referring not to ideal rationality as represented by theoretical models, but to simply the degree of rationality that people manifest in everyday inferences), and that is why it is a dysfunction when the capacity of such reasoning breaks down, as in severe psychotic states. (Wakefield, 1992b: 382)

The normal range will encompass instances of ‘ideal rationality’ (if they exist) but also the more realistic reasoning found across normal populations. Malfunctions which fall outside of that normal range will be the ones required for delivering a verdict of pathology. Let us see then if our etiological criterion which identifies *abnormal malfunction of belief* as necessary for doxastic pathology can prop up different judgements of pathology for conspiracy beliefs and delusions. In the next two sections we consider two possible loci for abnormal malfunction which could ground a judgement of pathology: provoking conditions (§3), and cognitive biases and deficits (§4). We argue for our primary conclusion that neither justify pathologizing delusions while not pathologizing conspiracy beliefs. Our discussion will support our secondary conclusion that the right response to this is to depathologize delusions.

## Provoking Conditions

We begin by considering conditions commonly thought to provoke delusional and conspiracy beliefs.

### Anomalous Experience and Epistemic Mistrust

As noted earlier, it is recognised that many delusions are preceded by highly anomalous experiences. Empiricist accounts of delusion formation have it that such experiences play a role in the genesis of delusional belief (in contrast to rationalism, which has it that experiences of this kind are downstream of delusional belief formation, see e.g. Campbell, 2001,12). The kinds of anomalous experiences subjects with delusions undergo might include ‘a discrepancy between intention and action’ (i.e. in delusions of alien control), and ‘hallucinations, substantial impairments of perceptual processes such as depth perception, the constancies of size, color, shape, etc., and the incapacity to exclude distracting input of various kinds’ (Maher 2003: 18). Some of the aforementioned experiences are positive in character, that is, they represent as present in the environment objects and properties which are in fact missing; but not all anomalous experiences associated with delusions are like this. Indeed, one of the more commonly discussed delusions, Capgras, is thought to be preceded by an experience of *absence* of expected affective response to a familiar face (Ellis & Young, 1990: 244). In Cotard delusion, it has been suggested that subjects do not experience any emotional feelings in response to their environment (Young et al., 1992: 800).

Can we identify something that plays a key prompting role in the formation of conspiracy beliefs, somewhat analogous to the role played by anomalous experiences in delusions? So far, research on conspiracy theories has been cautious in making claims concerning the *causal* contributions to the formation of the relevant beliefs, tending instead to make correlational claims about what might be typically *associated* with such beliefs (see e.g. Wood & Douglas, 2018; Douglas et al., 2019; Levy, 2019). However, there is broad consensus on at least some key contributions that are likely to play a causal role in the etiology of conspiracy beliefs – one of which is *epistemic mistrust*.

With this term we refer to a generalized suspicion towards authoritative informational accounts, experts’ opinions, and conventional wisdom, which many authors have identified as a first key step towards the endorsement of specific conspiracy theories and towards conspiracy mentality more generally (see e.g. Goertzl 1994; Abalakina-Paap et al., 1999; Uscinski and Parent 2014; Brotherton 2015: Ch. 5; Douglas et al., 2019; Levy, 2019; Van Prooijen 2019; Pierre, 2020; Brauner et al., 2023). It is a negative step, amounting to the rejection of an official view which is not deemed worthy of trust, or at least, the conviction that nothing is as it seems and appearances are often deceptive (Mayo, 2019: 147).

On Pierre’s (2020) influential two-component model of conspiracy belief, once this sort of mistrust takes hold, it creates an ‘epistemic vacuum’ that ‘asks to be filled’ by finding suitable alternatives – and it is here that the positive endorsement of particular conspiracy theories comes in. In a similar vein, Jan-Willem Van Prooijen argues that ‘a deep-rooted distrust in power-holders or other groups leads believers to reflexively reject official accounts of impactful events, and to uncritically accept implausible conspiracy theories’ (2019: 320). Felix Brauner and colleagues (2023) review a range of evidence which supports such a model – and in particular the role of epistemic mistrust within it.

As many authors have pointed out, the epistemic mistrust at play in the conspiracy mindset differs from paranoia (both clinical and sub-clinical) in important ways, and should be seen more as a social, rather than psychological, phenomenon. Paranoia is an eminently individual feeling, characterised by egocentricity and self-referentiality (the subject feels personally threatened), and by an intense fear due to the perception of the threat as ubiquitous (‘everyone is after me’). Insofar as it counts as paranoid, this fear is, by definition, excessive and ungrounded (Byford 2014: 124; Imhoff & Lamberty, 2017). Epistemic mistrust, by contrast, is a socially shared feeling of suspiciousness towards a specific target (powerful elites, official authorities), which arises from experiences of *collective* vulnerability, marginalization, and powerlessness (Abalakina-Paap et al., 1999; Douglas et al., 2019; Pierre, 2020). It is typically the result of the psychological dynamics underlying intergroup threats and the actual or perceived rejection by powerful outgroup members (van Prooijen and van Lange 2014: 245–6). As such, this sort of mistrust has a strong social dimension, meaning that the threat is not perceived at the personal level, but at the group level–and it is not (or at least not necessarily) ungrounded. Some authors have even argued that the epistemic mistrust at play in conspiracy theories may be an instance of healthy scepticism (see e.g. Coady, 2007).

### Anomalous Experience and Neurological Damage in Delusion

Our characterisation of epistemic mistrust suggests that it does not arise from abnormal malfunction, but rather is a not uncommon, and perhaps an understandable, even if not always rational, reaction to external threats (see Alper and Imhoff 2022). Anomalous experiences in delusion, on the other hand, sometimes arise from neurological damage. For example, in Capgras, the experience of reduced affective response to familiar faces is hypothesized to arise from ventromedial prefrontal cortex damage (Tranel, Damasio, and Damasio 1995; Coltheart, 2007). In the case of Cotard, it is hypothesized that subjects have a more generalized lack of affect and similar neurological damage is found. It might be thought that such damage is an abnormal malfunction, and so straightforwardly points to pathology – thereby marking a difference with conspiracy belief, where this damage (and indeed anything even approximating it) is absent.

However, there are two problems with identifying the locus of pathology in the anomalous experience associated with delusion. The first is that not all delusions are associated with anomalous experiences. Primary erotomania (the delusion that someone – usually of a higher status, e.g. a celebrity – is in love with one), is a delusion in which perfectly ordinary experiences are interpreted in such a way as to support the delusional belief. For example, license plates of cars of a certain type from a particular state, or the colour purple, are taken as messages of love (Jordan et al., 2006: 788). There is no straightforward case for pathology in experience here. We thus can’t justify the idea that delusions are pathological beliefs and conspiracy beliefs are not by appeal to experiential pathology because this is only present in a subset of delusions.^13^

Of course, it might be said that we need not identify a common locus of pathology across all delusions. It would nevertheless be of interest to identify the pathological character of a delusion with the experiential malfunction in those cases where it shows up. This leads us onto our second and more important point: we ought not move from experiential pathology to doxastic pathology.^14^ Abnormal malfunction elsewhere, does not provide grounds for positing pathology *of belief*. Crudely put, mechanisms of belief formation work with what they are provided from perceptual inputs, background beliefs, and so on. The production of a belief based on data resulting from experiential pathology does not make the resulting belief *itself* an instance of pathology. As Tim Bayne and Jordi Fernández say of empiricist accounts of delusion:

Although experience-based accounts conceive of delusions as grounded in psychological malfunction, they see that malfunction as restricted to experiential mechanisms, broadly construed; on their view, delusion involves no damage to the mechanisms of belief formation as such. (Bayne & Fernández, 2015: 6)

Pathology in experiential mechanisms which can give rise to strange experiences does not entail pathology in beliefs that result from those experiences. That is not to say that the resulting beliefs may not disrupt functioning to a significant degree, with severe impact on a person’s quality of life. Delusional beliefs absolutely have the potential to do that. But that is consistent with the idea that such problems do not arise as a result of abnormal malfunction of the mechanisms responsible for the formation of such beliefs.

In addition, there is evidence that the anomalous experiences associated with particular delusions do not give rise to delusions in all subjects (it is this empirical observation which is the motivation behind two-factor accounts of delusion to be discussed below, §4.2). The anomalous experience is present, but instead of it prompting a delusion, it prompts a belief of a different kind, for example, *I am ill,* or *that is my partner even though it doesn’t feel like her.* If we locate pathology in the anomalous experience, and take that to spread to the resultant belief, we would need to say that these non-delusional beliefs are also pathological. This is something we obviously ought not to say.

Of course there may be epistemically better and worse ways to respond to anomalous experience, and perhaps it will be said that not all responses to e.g. a lack of affective response to one’s partner, are reasonable ones. We agree, and nothing we say here prevents us evaluating various responses to anomalous experiences as better or worse than others. That evaluation though ought not to proceed by taking delusions to be pathological responses *on the grounds that* they are responses to experiences arising from pathology elsewhere. We must instead look more precisely to mechanisms of belief formation and evaluation as the site for doxastic pathology. Let us turn to do so now.

## The Role of Biases and Deficits

A range of biases and deficits have been hypothesized to be involved in delusions and conspiracy beliefs. Those interested in explaining the genesis and maintenance of these beliefs have taken markedly different approaches in this area. Research on conspiracy beliefs has proceeded by seeking to identify which normal range cognitive styles play an explanatory role, whilst much research on delusions (specifically, that in the service of defending the two-factor account) has sought to identify kinds of clinical irrationality to explain how such beliefs arise. We consider these approaches and argue that various purported second factors do not establish a malfunction in belief which could ground a judgement of doxastic pathology. Thus, the different approaches regarding doxastic pathology is not warranted, and since there are no grounds for doxastic pathology in the case of delusion, we ought to de-pathologize both phenomena.

### Cognitive Styles in Conspiracy Beliefs

It is commonplace in the research on conspiracy theories to observe that they are associated with a number of cognitive biases and reasoning fallacies (once again, many claims of this sort are correlational, but there are authors who have gone further, positing a proper causal influence of the biases upon the beliefs – see e.g. Brotherton 2015: 216, Pierre, 2020: 624–6). The biases in question are hypothesized to enter the scene when the subjects who mistrust and reject official explanations of some event search for alternatives. As we noted, such a search typically amounts to navigating through various (mis)information sources, deciding which ones to endorse. In so doing, it is hypothesized that subjects are driven by a number of information processing biases. Among the most widely recognized is the so-called *intentionality bias,* that leads us to favour explanations in which intentional agents play a key role and any relevant fact or event is seen as the result of deliberate intentional actions, rather than of mere coincidence, mechanical causes, or impersonal abstract forces (Brotherton & French, 2015; Douglas et al., 2016). A somewhat related bias is the *proportionality bias*, according to which ‘when big things happen, we look for big causes’ (Brotherton 2015: 211) – where the causes that we tend to see as ‘big’ are those involving the actions of intentional and powerful groups of agents (Ebel-Lam et al., 2010; Leman & Cinnirella, 2007). At a more general level, conspiracy theories have been described as driven by the basic *causality bias* that leads us to posit meaningful causal connections between co-occurring and spuriously correlated facts and events (van der Wal et al., 2018).

The formation of conspiracy beliefs may also be influenced by a variety of motivational factors. Explanations provided by conspiracy theories are cognitively appealing since they promise to satisfy important human needs. By leaving little room for randomness and tracing everything back to agential intentions, for instance, they promise to satisfy epistemic and existential needs like the *need for closure* and the *need for control* (Marchlewska et al., 2018; van Prooijen & Acker, 2015). By being unorthodox and non-mainstream, they promise to satisfy the so-called *need for uniqueness* – i.e. our desire to feel special and in possession of important information that is concealed to most (Imhoff & Lamberty, 2017; Lantian et al., 2017). By identifying external ‘culprits’ who can be blamed for difficult events which affect us, they promise to boost a positive image of the self and the group one belongs to (Cichocka et al., 2016). The list could continue. Whilst psychologists are careful to emphasise that it is not yet clear whether conspiracy beliefs do in fact satisfy the needs they *promise* to satisfy, there is nonetheless wide agreement that *seeking* to satisfy such needs is a basic motive that drives endorsement of such theories.

Importantly, whilst revealing some non-ideal rationality, these biases and motivational influences are not taken to result from abnormal malfunction. When researchers in this area use the term *bias,* they mean to pick out a cognitive style that is within the normal range for human psychology. That is, it is not a style that is exclusive to believers in conspiracy theories and it does not constitute a clinical abnormality:

[M]any of the cognitive biases and other psychological quirks that have been found to be associated with belief in conspiracy theories are universal, continuously distributed traits varying in quantity as opposed to all-or-none variables or distinct symptoms of mental illness. They are present in those who do not believe in conspiracy theories and some of them, like need for uniqueness or closure, may be valued or adaptive in certain culturally mediated settings. (Pierre, 2020: 618)

We see then that the cognitive styles associated with – and indeed contributory to – the formation of conspiracy beliefs are not ones which will warrant a classification of conspiracy beliefs as *pathological.* Whether these biases are instances of everyday malfunction is up for grabs – depending on whether they are best understood as heuristics selected by evolution, or simply as commonly and broadly distributed ways of going wrong. In any case, they are not abnormal malfunctions and so cannot ground a judgement of doxastic pathology. Let us now consider whether things are different in the case of delusions.

### Cognitive Styles in Delusion

Our discussion of biases in delusion naturally takes place within the context of the two-factor account of delusion, according to which anomalous experience is the first of two abnormalities to which we need to appeal in explaining delusion formation and/or maintenance. The second abnormality is to be located in some bias or deficit in belief formation or maintenance.

One of us has argued elsewhere that the two-factor account is unmotivated, and that the one-factor account ought to be considered the default approach for explaining monothematic delusions (Sullivan-Bissett, 2020, 2024b; Noordhof and Sullivan-Bissett 2021, 2023). Nevertheless, the two-factor approach probably represents the current orthodoxy, and we consider it here because it has sought to identify abnormalities which might be harnessed in an account of doxastic pathology (see e.g. Miyazono, 2015, 2018). We argue that various proposed second factors fail to establish doxastic pathology.

Two-factor theories come in three flavours: bias, deficit, and performance error. One version of the bias two-factor theory has it that people with delusions have a data-gathering bias which leads them to *jump to conclusions* (Garety & Freeman, 1999: 131). Another identifies the second factor as the tendency to privilege observational data over minimizing adjustments to one’s beliefs (Stone & Young, 1997). Talk of reasoning biases in the context of delusion formation can be understood in one of two ways. The first is to understand biases as ones which occur inside the normal range of intellectual styles, but tend to be associated with delusion. (This is equivalent to the sense of *bias* used in work on conspiracy belief formation discussed earlier.) If delusions are the result of normal intellectual styles applied to abnormal experiences, just like with the role of biases in conspiracy beliefs, we have no grounds for characterising them as pathological beliefs.

The second way to interpret bias talk is as identifying a reasoning bias not present in the ordinary population, or systematically worse in people with delusions. If two-factor approaches are read in this way, perhaps we would have a case for abnormal malfunction and, thus, doxastic pathology. However, the case for biases of this kind has not been made. For example, there is only some evidence of *the jumping to conclusions* bias in people with schizophrenia, but little evidence that it is present in the reasoning of those with monothematic delusions.^15^ Furthermore, there is some evidence that differences in reasoning between subjects with delusions and those without are not statistically significant (see e.g. McKay et al., 2007: 368–9; Brakoulisa et al. 2008: 157, 161–2; Jacobsen et al., 2012: 12) (see Noordhof & Sullivan-Bissett, 2021: 10286–92 for critical discussion of various bias theories along these lines).

Let us turn now to deficit versions of the two-factor approach which have focused on belief *evaluation* rather than *formation*. For example, Coltheart and colleagues have it that right hemisphere damage in the frontal lobe is responsible for malfunction in belief evaluation (Coltheart, 2007; Coltheart et al., 2007). The presence of such damage might be thought to establish doxastic pathology. However, it does not. Suppose that the damage was indeed in the regions of the brain responsible for belief evaluation. Nevertheless, we have not yet arrived at good grounds for claiming an *abnormal malfunction* in the mechanisms of belief evaluation. More needs to be said. We wouldn’t want to say, for example, that damage to a trait is sufficient for malfunction. That would mean cases of damage which maintained functional performance would nonetheless deliver a verdict of malfunction. Functional failure may often be proceeded by physical damage, but not always, and so the presence of physical damage to mechanisms of belief evaluation does not yet establish the malfunction we need to ground a judgement of doxastic pathology. For that, the damage would need to be responsible for a statistically abnormal failure of belief evaluation. Is there evidence for such a failure?

We do not think that there is, for two reasons. First, subjects with delusions are often able to make good judgements regarding the plausibility of their delusional beliefs (even if, at the same time, they are unable to abandon them). This is indicative of an ability to process information regarding a belief’s plausibility. In one case reported by Alexander and colleagues (1979), a subject with Capgras delusion was asked what he would think were someone to tell his story, in reply he said ‘I would find it extremely hard to believe’ (Alexander et al., 1979: 335). At least some people then, as Davies and colleagues point out, display ‘considerable *appreciation of the implausibility* of their delusional beliefs’ (Davies et al., 2001: 149). Of this case Philip Gerrans suggests of the subject that ‘[h]is grasp of the distinction between what is rationally required to believe in his context and what he actually believes is intact’ (Gerrans, 2001: 171). None of these observations *refute* the claim that the subject (and others like him) have a deficit in belief evaluation, but they are a little difficult to square with that claim. The deficit would have to be sufficiently robust as to affect the evaluation of one’s beliefs in the context of deliberating over whether to retain them, but also not so robust as to hinder one’s capacity for third-personal evaluations of their plausibility.

Second, at least some people with delusions do abandon their beliefs, which should not happen if the belief evaluation system is damaged.^16^ First-personal accounts of delusion abandonment are instructive here, since they often discuss the role of counterevidence in the abandonment (Flores, 2021: 6312–13). This counterevidence presumably played a role in a process of normal belief evaluation, which ought not to happen if there is a belief evaluation deficit. Again, we haven’t refuted the claim that subjects who abandon their delusion never had a deficit in belief evaluation – perhaps there was a deficit, and then subjects who abandoned their delusions *regained* the relevant capacities. However, if the deficit arises from neurological damage, this story is a harder sell. Overall then, the case for a failure of belief evaluation in delusion has not been made, let alone that failure being a statistically abnormal one.

Let us turn finally to performance error accounts. These are ones which rule out the characterisation of the second factor as one of bias or deficit. On such accounts, subjects with delusions have the capacity to, for example, form or evaluate beliefs appropriately, but there is a failure to put that capacity into practice (see e.g. Gerrans, 2001). The reason why the relevant capacity is inhibited will matter for judgements of pathology. We note that performance error theorists do not tend to appeal to any abnormal malfunction in mechanisms of belief formation in explaining the performance failure, but rather to contextual considerations. Thus, performance errors in delusion do not support a judgement of doxastic pathology.

We have considered various ways in which we might identify a pathology of belief by looking at two-factor theories of delusion. We have argued that bias theories will not give us pathology if they characterize the relevant biases as ones that are also present in non-delusional subjects, and have referred to work elsewhere critical of stronger versions of these views which could ground doxastic pathology. Deficit theories don’t yet give us pathology of *belief,* without evidence that belief evaluation is failing in a statistically abnormal way. Finally, performance error theories are simply not in the business of pathologizing delusion. Of course, none of this is to say that the work of mapping various cognitive styles associated with delusions isn’t important. We only claim that it is not work which can ground a judgement of pathology.

## Social Currency

The difference between delusional beliefs and conspiracy beliefs that we consider in this section has to do with the social side of belief formation and maintenance. Conspiracy beliefs have a strong social dimension: their themes are culturally recognisable, testimony plays a key role in their formation, they are typically shared by relatively large groups, and they can create and foster group bonding (see Bergamaschi Ganapini 2021; Ichino and Räikkä 2021). Indeed, there are whole communities of folk who are brought together by conspiratorial beliefs of certain kinds. By contrast, delusions have themes which are not culturally grounded, and they are generally not shared by same culture peers (this feature even makes it into the DSM-5’s *definition* of delusion). It might thus be thought legitimate to think in terms of pathology for delusions, because, although they share certain features with conspiracy beliefs (strange contents, resistance to counterevidence), they do not have the cultural or social support which might explain or excuse such features.

Indeed, in reflecting on the culture clause in the DSM-5’s definition of delusion, Dominic Murphy says:

Our expectations about normal belief formation include expectations about the kinds of beliefs people are likely to acquire in the course of normal human development. […] we think that it is normal for people to pick up beliefs that we find weird from the culture around them, and not normal for them to arrive at equivalently weird beliefs all by themselves in cultures that provide no support for such beliefs. (Murphy, 2013: 119)

Here it might be thought that any differences with respect to cultural and social currency are only relevant to our argument insofar as they are related to the presence or absence of abnormal malfunction in mechanisms of belief formation. Since there is no such relation, we need not consider these differences.

Although we agree that this route is available to us, we also suspect that the differences in question are likely to arise in readers’ minds. Indeed, it may strike one as sufficiently serious that if these differences are not relevant to the characterisation of doxastic pathology we’re adopting, then all the worse for our characterisation of doxastic pathology! We agree with Murphy that the relationship between belief and culture is important, and so too is a belief having social support: this is why we speak to these differences as they show up in conspiracy beliefs and delusions.

We make three points in response to the observation that conspiracy beliefs enjoy significant cultural and social currency and delusions do not: (1) the relationship between delusional content on the one hand, and culture on the other, is not as absent as might be thought; (2) the testimonial differences between conspiracy beliefs and delusions may be less sharp than they are often taken to be; and (3) even if appeals to social currency can play a significant role in defending conspiracy beliefs from charges of doxastic pathology, the nature of anomalous experience can perform a similar role with respect to delusions.

### Delusion and Culture

There are of course cultural influences on delusional content, and in at least some cases, the bizarreness and ununderstandability of delusional contents has been overstated. As Bortolotti points out, the Capgras delusion:

reminds us of science-fiction stories where clones or cleverly disguised aliens gradually replace the people around us – and none is the wiser apart from us. It then becomes a challenge to persuade everybody else that the gradual substitutions are indeed happening. (Bortolotti, 2023: 41)

Not only do we find Capgras themes in fiction, there are also news reports or rumours regarding people being replaced by look-alikes (Avril Lavigne, Paul McCartney, Kate Middleton, and Melania Trump have all recently been the subjects of such stories across the media). More generally, Gold and Gold have argued that a key characteristic of delusions is *cultural sensitivity,* that is, ‘at least some delusions are highly sensitive to cultural context, and the details of delusional ideas vary as culture does’ (Gold and Gold 2024: 534).

Admittedly, it is one thing for the content of a belief to have a cultural origin or parallel, it is quite a different thing for the content of a belief to be shared across thousands of one’s peers. That is, although delusional contents enjoy some cultural support which might be taken by an individual to speak to the possibility or probability of the content, they do not have the much broader social currency enjoyed by conspiracy beliefs. And so someone might still insist that this is what justifies not pathologizing conspiracy beliefs, whilst pathologizing delusions. Conspiracy beliefs’ bizarre contents and imperviousness to counterevidence can be *excused* by the social currency that such beliefs enjoy. While, on the other hand, even if delusions have more cultural grounding than they are typically credited with, the fact that they are *not* shared by same culture peers (by definition) means that in this case we have a bizarre belief, impervious to counterevidence, for which we cannot make a similar excuse. Indeed, perhaps delusions’ idiosyncratic character indicates doxastic malfunction – even if it falls short of establishing its nature. Whilst social currency might bring conspiracy beliefs back from the brink of pathological status, delusional beliefs are left teetering.

### The Role of Testimony in Delusions and Conspiracy Beliefs

It might be thought that there are significant testimonial differences between conspiracy beliefs and delusional beliefs: most obviously, the former enjoy testimonial support that the latter do not (indeed, delusions persist *in spite of* testimony against them).^17^ However, the alleged differences in this respect are, in fact, less sharp than often presumed.

Consider first the relationship between testimony and delusions, recently explored by Miyazono and Alessandro Salice (2021).^18^ We might ask, as they do: why is the subject with a delusion responsive to some kinds of evidence (that presented in anomalous experience) but not to other kinds (the testimony of others speaking to the implausibility of her belief)? Miyazono and Salice argue that this ‘rather puzzling pattern’ can be explained by their proposal that the subject ‘responds to different kinds of evidence in different ways’ (2021: 1840). In particular, the subject is *especially irresponsive* to testimonial evidence, but has no such problem when it comes to experiential evidence. To explain why testimony is not given the credibility it is due in delusion, Miyazono and Salice talk of testimonial *abnormalities,* identifying two of them in particular: *testimonial isolation* (a lack of testimonial interactions with others), and *testimonial discount* (an arbitrarily selective neglect of testimony from others). If understood as tracking doxastic malfunction, these two abnormalities might be thought to provide a new route to pathology for delusion but not for conspiracy beliefs.

However, this privileging of one’s first personal evidence and failing to take on board that offered within testimony from others is a fairly everyday occurrence, it does not, we say, represent a ‘puzzling pattern’. The situation may be akin to someone self-deceived as to the nature of her romantic relationship: she may acknowledge that the facts indicate that her partner is abusive and she may trust testimonies about his bad reputation, yet fail to form the relevant beliefs that this is so due to some dimension of her first-person experience (see discussion in Noordhof, 2003).

Furthermore dismissal of testimony clearly also occurs, to some extent, in the context of conspiracy beliefs, which are, by definition, minority beliefs held in the face of substantial and credible conflicting testimony. At least at a collective level, conspiracy theories’ endorsement is the product of so-called ‘testimonial abnormalities’ similar to those that Miyazono and Salice take to be causally relevant in the formation of delusions. Specifically, the ‘epistemic bubbles’ and ‘echo chambers’ that contribute to the formation and spreading of conspiracy beliefs (Nguyen 2020) are the equivalent at the group level of Miyazono and Salice’s ‘testimonial isolation’ and ‘testimonial discount’ in delusions. In this respect, notwithstanding the local peer testimonial support conspiracy beliefs enjoy, they nevertheless display an imperviousness to testimony more broadly analogous to the one displayed by delusions.

### Anomalous Experience Again

As we have seen, subjects with delusions often have striking, repeated, and intense anomalous experiences. We suggest that the profundity and specificity of such experiences might compensate for the absence of social support enjoyed by delusions.^19^ So let us accept that delusions face an absence of testimonial support from one’s peers, and do not enjoy the benefits that come with holding in-group beliefs (see Williams, 2021). Nevertheless, they are formed in a highly charged context of intense, often distressing, confusing, identity-threatening, experiences. So yes, conspiracy beliefs may enjoy some peer support, but the first-personal evidence on the basis of which they are formed is substantially less profound. Epistemic mistrust presents a far flimsier evidence base than the anomalous experiences subjects with delusions are responding to.

Of course, we have no formal calculus to offer which would demonstrate that the swing to judgement of doxastic pathology encouraged by delusions’ weaker social support is compensated by reflection on the nature of the anomalous experiences to which people who form delusions are responding. We must be more modest than that. But we hope that our discussion encourages suitable modesty from those who take it that appeal to social currency is a slam dunk against any claim which seeks to bring delusions onto a continuum with other beliefs with respect to doxastic pathology. With appropriate philosophical modesty, then, we suggest that if there is a gap to be mitigated left by the absence of social support, it can be filled by thinking carefully about the nature of anomalous experiences which prompt delusions.

In sum, considerations regarding cultural and social currency do not justify the claim that conspiracy beliefs are a normal range phenomenon whilst delusions involve doxastic pathology.

## Conclusions

We have argued that the different approaches taken to delusions and conspiracy beliefs with respect to their pathological status is unwarranted. We began by showing that whilst conspiracy beliefs are typically taken to be a normal range phenomenon, delusional beliefs are typically taken to be instances of pathology. We considered standard accounts of provoking conditions and of the role of biases and deficits in these phenomena, as well as differences between them in cultural and social currency. We argued that none justify taking conspiracy beliefs to be a normal range phenomenon while taking delusions to be pathological beliefs. The right response to this is to de-pathologize both phenomena.

What we think is interesting about the direction of travel in conspiracy research is that it does not seek to explain conspiracy theories by any abnormal way of forming beliefs, whereas research in delusion does the opposite. Our view is that research on delusion would do well to frame itself in a way similar to research on conspiracy theories–as a search for normal range cognitive styles associated with particular kinds of belief. In any case, we have argued that neither conspiracy beliefs nor delusions involve the sort of malfunction in belief required for doxastic pathology.

## Footnotes

1 See for example David Connett (2021) in *The Guardian,* Rebecca Ruiz (2021) in *Mashable,* and David Shearman (2018) on *ABC News.*

2 The resistance to pathologizing conspiracy beliefs is also present in disciplines beyond psychology and philosophy. For example, Michael Butler and Peter Knight note that ‘[t]he cultural studies approach […] explicitly rejects the pathologizing tendency’ (2020: 31).

3 These approaches are often captured under the labels of *generalist* and *particularist* (see Boudry and Napolitano 2023 for scepticism about the usefulness of this way of carving the theoretical joints).

4 In particular, two-factor theories of delusion (which we discuss later) were originally motivated by consideration of monothematic delusions, and proponents continue to restrict the remits of their accounts in this way (see e.g. Davies et al., 2001: 137, Coltheart, Langdon, and McKay 2011: 282, Coltheart 2013: 103, Coltheart and Davies 2021: 225–6). Delusions occurring in the context of some broader disorder (e.g. polythematic delusions in schizophrenia) might be better candidates for instances of pathological belief (even if the complexity of the broader condition makes identifying specific sites of pathology challenging).

5 Successful challenges to doxasticism in either case may change the landscape of our discussion. Challenges to doxasticism for delusions include those from G. E. Berrios (1991), Gregory Currie (2000), Andy Egan (2008), and Richard Dub (2017) (see Noordhof 2024 for critical discussion). Challenges to doxasticism for conspiracy attitudes include Anna Ichino (2022), Ichino and Juha Räikkä (2021), Marianna B. Ganapini (2022), and Neil Levy (2024). While recognizing that such challenges might undermine at least part of our arguments, we make two quick points. First, non-doxasticism does not typically involve a universal claim: non-doxasticists do not argue that their target attitudes are *never* beliefs, but only that some of them are not (see e.g. Currie 2000 for delusions; Ichino 2022 for conspiracy beliefs). Thus our arguments would still hold at least for those delusions and conspiracy attitudes for which a doxastic account is appropriate. Second, non-doxasticism per se is a negative view which says that delusions and conspiracy attitudes are *not* (always) beliefs, while leaving open the positive question of what type of attitude they are. If they turned out to be the same type of non-doxastic attitude, for example, imaginings (see again Currie 2000 for delusions; Ichino 2022 for conspiracy attitudes), a suitably adapted version of our argument might still hold.

6 Even if cases of delusions and conspiracy beliefs involving doxastic pathology are not the normal case, that does not speak against their importance. Rather, insofar as our argument is correct, it becomes crucial to understand which factors and conditions may contribute to doxastic pathology in these particular cases.

7 This is not to say that the only beliefs apt for clinical intervention are those involving doxastic pathology. In what follows we understand pathology as arising from *malfunction* and although it has been argued in the case of delusions that only those involving malfunction are appropriate targets of intervention (Sakakibara 2016), we agree with Peter Clutton and Stephen Gadsby (2018) that something’s arising from normal functioning ought not rule it out as a candidate for treatment. Nevertheless, if we are in the business of treating delusions which arise from pathology, we take it that it is important to identify the locus of that pathology.

8 Another way into characterising delusions as especially epistemically problematic is via hypothesis selection. If delusions are explanations of anomalous experience, it might be asked why subjects pick explanations which are hugely implausible, or indeed, ‘nonstarters’ (Fine et al., 2005: 160). Some have even suggested that we should posit impaired or disrupted mechanisms of hypothesis generation (Parrott 2021: 342). However, constructing implausible hypotheses is not unique to delusions (conspiracy beliefs and paranormal beliefs are in the same boat), and so cannot provide the grounds for liberating a judgement of pathology based on epistemic considerations.

9 See Levy (2022) for a departure from this consensus regarding the epistemic status of conspiracy beliefs.

10 The idea of delivering epistemic benefits that are otherwise unavailable is key to the notion of *epistemic innocence.* For more on delusion and epistemic innocence see Bortolotti (2015, 2016) and Sullivan-Bissett (2018).

11 Of course in some populations functional abnormalities are nonetheless statistically normal (such as short-sightedness). This represents an issue for a general account of pathology based on functional abnormalities against a statistical assumption. We don’t take this definition to be a happy one across the piece, but it will serve for the purposes of our discussion where the abnormalities picked out by two-factor theories (to be discussed shortly) are taken to be both functional and statistical abnormalities.

12 This view, which has it that delusions arise from *organic malfunction* resulting in a ‘top-down disturbance in the subject’s beliefs’ (Campbell 2001: 91), might be a natural ally of the claim that delusions arise from doxastic pathology. Our discussion here, however, takes place against the background of the more orthodox bottom-up, empiricist framework about delusions, and so we don’t discuss this possibility any further.

13 A potential reply to this point is that some accounts of erotomania retain continuity with other delusions by hypothesizing that it arises from an erroneous attribution of salience to events in experience (Coltheart 2010: 24–5). If that’s right, we might then regain the claim that delusions involve a pathology in experience, and not run into the problem.

14 This point is also stressed by those attracted to the idea that delusions might be *adaptive responses* (see for example discussion in Garson 2022: 259–260).

15 In addition, it has been found that the relationship between holding delusion-like beliefs and jumping to conclusions is ‘severely attenuated’ or ‘disappeared entirely’ when participants coded as ‘careless’ are removed from the data (Sulik et al. 2023: 757, see also Ross et al., 2017, and Bayne 2017: 81 for discussion of the bias as it occurs in non-delusional populations).

16 In addition, some cases of monothematic delusion do not present with this damage. And so, like we discussed earlier with regard to anomalous experience, the idea that delusions are pathological beliefs (and conspiracy beliefs are not) cannot be made by appeal to this damage since it is only present in a subset of delusions.

17 This observation could be framed in Bayesian terms, noting that conspiracy beliefs can be seen as rationally arising in response to testimony via Bayesian mechanisms in a way in which delusions cannot. Here we do not work within a Bayesian framework, but we take some of the discussion which follows to speak to this idea. We’re grateful to an anonymous reviewer for raising this point.

18 Although their discussion takes place in the context of delusions in schizophrenia, we can see that insofar as it has application, it has so in our target cases too.

19 As noted earlier (§3.2), there are some cases where subjects have anomalous experiences often associated with delusion, but do not themselves become delusional. This might suggest that they are not so compelling after all, since not everyone is compelled by them. Brendan Maher (1999) and B. Lana Frankle (2021) have argued that where these experiences do not compel one to delusion, that’s because they lack in some way (perhaps they’re less intense or less systematic). We are not committed to this (for discussion see Noordhof anf Sullivan-Bissett, 2021, sect. 5; Sullivan-Bissett, 2024b, sect. 5). Nevertheless, we think that experiences can be *profound* without *compelling* one to a particular belief.

## References

[CR1] Abalakina-Paap, M., Stephan, W. G., Craig, T., & Gregory, W. L. (1999). Beliefs in conspiracies. *Political Psychology,**20*, 637–647.

[CR2] Alexander, M. P., Stuss, D. T., & Benson, D. F. (1979). Capgras’ syndrome: A reduplicative phenomenon. *Neurology,**29*, 334–339. 571979 10.1212/wnl.29.3.334

[CR3] Alper, S., and Imhof, R. (2022). Suspecting foul play when it is objectively there: The association of political orientation with general and partisan conspiracy beliefs as a function of corruption levels. *Social Psychological and Personality Science*, 1–11.

[CR4] American Psychiatric Association (2000). 2013 diagnostic and statistical manual of mental disorders, 4th Edn, 5th Edn.

[CR5] Bayne, T. (2017). Delusion and the norms of rationality. *Rationality* pp. 77–94. Elsevier.

[CR6] Bayne, T., & Fernández, J. (2015). Delusion and self-deception: mapping the terrain. In T. Bayne & J. Fernández (Eds.), *Delusion and Self-deception* pp. 1–21. Psychology Press.

[CR8] Bergamaschi Ganapini, M. (2021). The signaling function of sharing fake stories. *Mind & Language,**38*(1), 64–80.

[CR200] Bergamaschi Ganapini, M. (2022). Absurd stories, ideologies and motivated cognition. *Philosophical Topics, 50*(2), 21–39.

[CR9] Berrios, G. E. (1991). Delusions as “Wrong Beliefs”: A conceptual history. *British Journal of Psychiatry,**159*, 6–13. 1840782

[CR11] Bortolotti, L. (2009). *Delusions and Other Irrational Beliefs*. Oxford: Oxford University Press

[CR12] Bortolotti, L. (2015). *The epistemic innocence of motivated delusions*. *Consciousness and Cognition,**33*, 490–499. 25459652 10.1016/j.concog.2014.10.005 PMC7618694

[CR13] Bortolotti, L. (2016). Epistemic benefits of elaborated and systematized delusions in schizophrenia. *British Journal for the Philosophy of Science,**67*(3), 879–900. 27924116 10.1093/bjps/axv024 PMC4990704

[CR14] Bortolotti, L. (2018). ‘Delusion’. The Stanford Encylopedia of Philosophy. Spring 2018 Edition. Zalta, Edward (ed). https://plato.stanford.edu/entries/delusion/.

[CR15] Bortolotti, L. (2020). *The Epistemic Innocence of Irrational Beliefs*. Oxford University Press.

[CR16] Bortolotti, L. (2022). ‘Are Delusions Pathological?’ *Asian Journal of Philosophy*, *33*(1)

[CR17] Bortolotti, L. (2023). *Why delusions matter*. Bloomsbury.

[CR18] Bortolotti, L., Gunn, R., and Sullivan-Bissett, E. (2017). ‘What makes a belief delusional?’ In Mac Carthy, Ita, Sellevold, Kirsti, and Smith, Olivia (Eds) *Cognitive Confusions: Dreams, Delusions and Illusions in Early Modern Culture* pp. 37–51. Legenda.

[CR19] Boudry, M., & Napolitano, M. G. (2023). Why we should stop talking about generalism and particularism. Moving the debate about conspiracy theories forward. *Social Epistemology Review and Reply Collective,**2*, 22–26.

[CR20] Brakoulias, V., Langdon, R., Sloss, G., Coltheart, M., Meares, R., & Anthony, H. (2008). Delusions and reasoning: A study involving cognitive behavioural therapy. *Cognitive Neuropsychiatry,**13*(2), 148–165. 18302027 10.1080/13546800801900587

[CR21] Brauner, F., Fonagy, P., Campbell, C., Griem, J., Storck, T., & Nolte, T. (2023). Trust Me: Don’t Trust Anyone: How epistemic mistrust and credulity are associated with conspiracy mentality. *Research in Psychotherapy,**26*, 705. 38156557 10.4081/ripppo.2023.705 PMC10782893

[CR22] Brotherton, R. (2015). *Suspicious Minds. Why we Believe Conspiracy Theories*. Bloomsbury Sigma.

[CR23] Brotherton, R., & French, C. C. (2015). Intention seekers: Conspiracist ideation and biased attributions of intentionality. *PLoS ONE,**10*(5), e0124125. 25970175 10.1371/journal.pone.0124125 PMC4430300

[CR24] Butler, M. and Knight, P. (2020). Conspiracy theory in historical, cultural and literary studies. In Butler, M. and Knight, P. (Eds.). *Routledge Handbook of Conspiracy Theories*. Routledge (pp. 28–42)

[CR25] Byford, Jovan (2014). *Conspiracy Theories. A Critical Introduction*. Palgrave Macmillan.

[CR26] Cassam, Q. (2019). *Conspiracy Theories*. John Wiley.

[CR27] Campbell, J. (2001). Rationality, meaning, and the analysis of delusion. *Philosophy, Psychiatry & Psychology,**8*, 89–100.

[CR28] Cichocka, A., Marchlewska, M., & Golec de Zavala, A. (2016). Does self-love or self-hate predict conspiracy beliefs? Narcissism, self-esteem, and the endorsement of conspiracy theories. *Social Psychological and Personality Science,**7*, 157–166.

[CR29] Clutton, P., & Gadsby, S. (2018). Delusions, harmful dysfunctions, and treatable conditions. *Neuroethics,**1*, 167–181.

[CR30] Coady, D. (2007). Are conspiracy theories irrational? *Episteme,**1*, 193–204.

[CR31] Coltheart, M. (2007). Cognitive neuropsychology and delusional belief. *The Quarterly Journal of Experimental Psychology,**60*(8), 1041–1062. 17654390 10.1080/17470210701338071

[CR32] Coltheart, M. (2010). The neuropsychology of delusions. *Annals of the New York Academy of Sciences,**1191*, 16–26. 20392273 10.1111/j.1749-6632.2010.05496.x

[CR33] Coltheart, M. (2013). On the distinction between monthematic and polythematic delusions. *Mind & Language,**28*(1), 103–112.

[CR34] Coltheart, M. and Davies, M. (1991). *Pathologies of Belief.* Wiley-Blackwell.

[CR35] Coltheart, M., & Davies, M. (2021). Failure of hypothesis evaluation as a factor in delusional belief. *Cognitive Neuropsychiatry,**26*(4), 213–260. 33874847 10.1080/13546805.2021.1914016

[CR36] Coltheart, M., Langdon, R., & McKay, R. (2007). Schizophrenia and monothematic delusions. *Schizoprenia Bulletin,**33*(3), 642–647. 10.1093/schbul/sbm017 PMC2526128 17372282

[CR37] Coltheart, M., Langdon, R., & McKay, R. (2011). Delusional belief. *Annual Review of Psychology,**62*(1), 271–298. 10.1146/annurev.psych.121208.131622 20731601

[CR38] Connett, D. (2021). Naomi wolf banned from twitter for spreading vaccine myths. *The Guardian.* Accessed Oct 2021. https://www.theguardian.com/books/2021/jun/05/naomi-wolf-banned-twitter-spreading-vaccine-myths.

[CR39] Currie, G. (2000). Imagination, hallucination and delusion. *Mind & Language,**15*, 168–183.

[CR40] Davies, M., Coltheart, M., Langdon, R., & Breen, N. (2001). Monothematic delusions: towards a two-factor account. *Philosophy, Psychiatry, & Psychology,**8*(2–3), 133–158.

[CR41] Dentith, M. (2014). *The Philosophy of Conspiracy Theories*. Palgrave Macmillan.

[CR42] Douglas, K. (2021). Are conspiracy theories harmless? *The Spanish Journal of Psychology,**24*, 1–7. 10.1017/SJP.2021.10 33612140

[CR43] Douglas, K., Uscinski, J., Sutton, J., Cichocka, A., Nefes, T., Siang Ang, C., & Deravi, F. (2019). Understanding conspiracy theories. *Political Psychology,**40*(S1), 3–35.

[CR44] Douglas, K. M., Sutton, R. M., Callan, M. J., Dawtry, R. J., & Harvey, A. J. (2016). Someone is pulling the strings: Hypersensitive agency detection and belief in conspiracy theories. *Thinking & Reasoning,**22*(1), 57–77.

[CR45] Dub, R. (2017). Delusions, acceptances, and cognitive feelings. *Philosophy and Phenomenological Research,**94*(1), 27–60.

[CR46] Duetz, J. (2022). Conspiracy theories are not beliefs. Erkenntnis. 1–15.

[CR47] Ebel-Lam, A., Fabrigar, R., MacDonald, T., & Jones, S. (2010). Balancing causes and consequences: The magnitude-matching principle in explanations for complex social events. *Basic and Applied Social Psychology,**32*(4), 348–359.

[CR48] Egan, A. (2008). Imagination, delusion, and self-deception. In T. Bayne and J. Fernandez (Eds.) *Delusion and Self-Deception.* Psychology Press, pp. 263–80

[CR49] Ellis, H. D., & Young, A. (1990). Accounting for delusional misidentifications. *British Journal of Psychiatry,**157*, 239–248. 10.1192/bjp.157.2.239 2224375

[CR50] Fine, C., Craigie, J., & Gold, I. (2005). The explanation approach to delusion. *Philosophy, Psychiatry, and Psychology,**12*(2), 159–163.

[CR51] Flores, C. (2021). Delusional evidence-responsiveness. *Synthese,**199*(3–4), 6299–6330.

[CR52] Frankle, B. S., & Lana. (2021). In defence of the one-factor doxastic account: A phenomenal account of delusions. *Consciousness and Cognition,**94*, 10381. 10.1016/j.concog.2021.103181 34418637

[CR53] Garety, P., & Freeman, D. (1999). Cognitive approaches to delusions: A critical review of theories and evidence. *British Journal of Clinical Psychology,**38*, 113–154. 10389596 10.1348/014466599162700

[CR54] Garson, J. (2022). *Madness: a Philosophical Exploration*. New York: Oxford University Press.

[CR55] Gerrans, P. (2001). Delusions as performance failures. *Cognitive Neuropsychiatry,**6*(3), 161–173. 16571516 10.1080/1354680004200016

[CR56] Gold, I. and Gold, J. (2024). Delusion and culture. In Sullivan-Bissett, Ema (ed.). *The Routledge Handbook of the Philosophy of Delusion*. Oxon: Routledge, pp. 533–543.

[CR57] Haidt, J. (2013). *The Righteous Mind*. Westminster: Penguin Books Ltd.

[CR58] Hofstadter, R. (1964). *The Paranoid Style in American Politics*, Harper’s Magazine

[CR59] Ichino, A. (2022). Conspiracy theories as walt-fiction. In L. Julia and E. Patrik (Eds.). *The Philosophy of Fiction. Imagination and Cognition*. Routledge, 240–261.

[CR554] Ichino, A. (2024). What is a conspiracy theory (and why is this an important question)?' *Rivista di filosofia,**2*, 281–302.

[CR60] Ichino, A., & Räikkä, J. (2021). Non-doxastic conspiracy theories. *Argumenta,**7*(1), 247–263.

[CR61] Imhoff, R., & Lamberty, P. K. (2017). Too special to be duped: Need for uniqueness motivates conspiracy beliefs. *European Journal of Social Psychology,**47*, 724–734.

[CR62] Jacobsen, P., Freeman, D., & Salkovskis, P. (2012). Reasoning bias and belief conviction in obsessive-compulsive disorder and delusions: Jumping to conclusions across disorders? *British Journal of Clinical Psychology,**51*(1), 84–99. 22268543 10.1111/j.2044-8260.2011.02014.x

[CR63] Jolley, D., & Paterson, J. L. (2020). ‘Pylons Ablaze: Examining the role of 5C COVID-19 conspiracy beliefs and support for violence. *British Journal of Social Psychology,**59*, 628–640. 32564418 10.1111/bjso.12394 PMC7323354

[CR64] Jordan, H. W., Lockert, E. W., Johnson-Warren, M., Cabel, C., Cooke, T., Greer, W., & Howe, G. (2006). Erotomania revisited: Thirty Four Years later. *Journal of the National Medical Association,**98*(5), 787–793. 16749657 PMC2569288

[CR65] Lantian, A., Muller, D., Nurra, C., & Douglas, K. M. (2017). “I know things they don’t know!” the role of need for uniqueness in belief in conspiracy theories. *Social Psychology,**48*, 160–173.

[CR66] Leman, P., & Cinnirella, M. (2007). A major event has a major cause: Evidence for the role of heuristics in reasoning about conspiracy theories. *Social Psychology Review,**9*, 18–28.

[CR67] Levy, N. (2019). Is conspiracy theorising irrational? *Social Epistemology Review and Reply Collective,**8*, 65–76.

[CR68] Levy N. (2022). *Bad Beliefs. Why They Happen to Good People*. Oxford University Press. 35167201

[CR70] Levy, N. (2024). Believing in Shmeliefs. *Ergo,**11*(18), 466–488.

[CR71] Maher, B. (1999). Anomalous experience in everyday life: its significance for psychopathology. *The Monist,**82*(4), 547–570.

[CR72] Maher, B. (2003). Schizoprenia, aberrant utterance and delusions of control: The disconnection of speech and thought, and the connection of experience and belief’. *Mind & Language,**18*(1), 1–22.

[CR73] Maher, B. (2006). The relationship between delusions and hallucinations. *Current Psychiatry Reports,**8*, 179–183. 19817067 10.1007/s11920-006-0021-3

[CR74] Mayo, R. (2019). The skeptical (Ungullible) mindset. In F. Joseph and B. Roy (Eds.). *The Social Psychology of Gullibility. Fake News, Conspiracy Theories, and Irrational Beliefs.* Oxford University Press (pp. 140–158) Routledge.

[CR75] Marchlewska, M., Cichocka, A., & Kossowska, M. (2018). Addicted to answers: Need for cognitive closure and the endorsement of conspiracy beliefs. *European Journal of Social Psychology,**48*(2), 109–117.

[CR76] McKay, R., Langdon, R., & Coltheart, M. (2007). Jumping to Delusions? Paranoia, probabilistic reasoning, and the need for closure. *Cognitive Neuropsychiatry,**12*(4), 362–376. 17558643 10.1080/13546800701203769

[CR77] McKenna, P. (2017). *Delusions: Understanding the un-understandable*. Cambridge: Cambridge University Press.

[CR78] Miyazono, K. (2015). Delusions as harmful malfunctioning beliefs. *Consciousness and Cognition,**33*, 561–573. 25467777 10.1016/j.concog.2014.10.008

[CR79] Miyazono, K., & Salice, A. (2021). Social epistemological conception of delusion. *Synthese,**199*, 1831–1851.

[CR81] Murphy, D. (2013). Delusions, modernist epistemology and irrational belief. *Mind & Language,**28*(1), 113–124.

[CR82] Napolitano, M. Giulia. (2021). Conspiracy theories and evidential self-insulation. In S. Bernecker et al. (Eds). *The Epistemology of Fake News* (pp. 82–106) Oxford University Press.

[CR83] Noordhof, P. (2003). Self-deception, interpretation and consciousness. *Philosophy and Phenomenological Research,**57*(1), 75–100.

[CR84] Noordhof, P. (2024). Delusion and non-Doxasticism. In E. Sullivan-Bissett (Ed.), *The Routledge Handbook of Philosophy of Delusion* (pp. 308–323). Routledge.

[CR85] Noordhof, P., & Sullivan-Bissett, E. (2021). The clinical significance of anomalous experience in the explanation of monothematic delusions. *Synthese,**199*, 10277–10309.

[CR86] Noordhof, P. and Sullivan-Bissett, E. (2023). The everyday irrationality of monothematic delusion. In P. Henne, and S. Murray, eds.) *Advances in Experimental Philosophy of Action*. (pp. 87–111) Bloomsbury.

[CR87] Nyguyen, T. C. (2020). Echo chambers and epistemic bubbles. *Episteme,**17*(2), 141–161.

[CR88] Parrott, M. (2021). Delusional predictions and explanations. *The British Journal for the Philosophy of Science,**72*(1), 325–353.

[CR89] Petrolini, V. (2017). What makes delusions pathological? *Philosophical Psychology,**30*(4), 502–523.

[CR90] Petrolini, V. (2024). Delusion and pathology. In E. Sullivan-Bissett, (ed.). *The Routledge Handbook of Philosophy of Delusion*. Oxon: Routledge, pp. 33–45.

[CR91] Pierre, J. M. (2020). Mistrust and misinformation: A two-component, socio-epistemic model of belief in conspiracy theories. *Journal of Social and Political Philosophy,**8*(2), 617–641.

[CR92] Radnitz, S., & Underwood, P. (2015). Is belief in conspiracy theories pathological? *A Survey Experiment on the Cognitive Roots of Extreme Suspicion’, British Journal of Political Science,**47*, 113–129.

[CR93] Ritunnano, R., & Bortolotti, L. (2022). Do delusions have and give meaning? *Phenomenology and the Cognitive Sciences,**21*, 949–968. 36034162 10.1007/s11097-021-09764-9 PMC9399029

[CR94] Ross, R. M., Pennycook, G., McKay, R., Gervais, W. M., Langdon, R., & Coltheart, M. (2016). Analytic cognitive style, not delusional ideation, predicts data gathering in a large beads task study. *Cognitive Neuropsychiatry,**21*(4), 300–314. 27341507 10.1080/13546805.2016.1192025

[CR95] Ruiz, R. (2021). Conspiracy theories are a mental health crisis. *Mashable.* Accessed Oct 2021: https://mashable.com/article/mental-health-disinformation-conspiracy-theories-depression?fbclid=IwAR3Y3wDi-JpvdKj-K6iqByT30iL50nr7TW7jbs5kFfoMz2DmvWyChWEAzCY.

[CR96] Sakakibara, E. (2016). Irrationality and pathology of beliefs. *Neuroethics,**9*, 147–157.

[CR98] Shearman, D. (2018). Climate change denial is delusion, and the biggest threat to human survival. *ABC News*. Accessed Oct 2021. https://www.abc.net.au/news/2018-12-07/climate-change-denialism-holocaust-david-attenborough-coal/10585744.

[CR201] Sulik, J., Ross, R. M., Balzan, R., & McKay, R. (2023). Delusion-like beliefs and data quality: Are classic cognitive biases artifacts of carelessness? *Journal of Psychopathology and Clinical Science, 132*(6), 749–760. 10.1037/abn0000844 37326560

[CR99] Stone, T., & Young, A. W. (1997). Delusions and brain injury: the philosophy and psychology of belief. *Mind and Language,**12*(3–4), 327–364.

[CR202] Sullivan-Bissett, E. (2018). Monothematic delusion: A case of innocence from experience. *Philosophical Psychology,**31*(6), 920–947.

[CR588] Sullivan-Bissett, E. (2020). Unimpaired abduction to alien abduction: Lessons on delusion formation. *Philosophical Psychology,**33*(5), 679–704.

[CR100] Sullivan-Bissett, E. (2024a). Introduction. In E. Sullivan-Bissett (Ed.), *The Routledge Handbook of Philosophy of Delusion* (pp. 1–29). Routledge.

[CR203] Sullivan-Bissett, E. (2024b) The one-factor account. In E. Sullivan-Bissett (Ed.), *The Routledge Handbook of Philosophy of Delusion*. (pp. 414–429). Oxon: Routledge.

[CR101] Sunstein, C. R., & Vermuele, A. (2009). Conspiracy theories: Causes and cures. *Journal of Political Philosophy,**17*, 202–227.

[CR102] Tranel, D., Damasio, H., & Damasio, A. R. (1995). Double dissociation between overt and covert face recognition. *Journal of Cognitive Neuroscience,**7*(4), 425–432. 23961902 10.1162/jocn.1995.7.4.425

[CR103] Uscinski, J. E. (Ed.), (2018). *Conspiracy Theories and the People Who Believe Them*, Oxford University Press.

[CR104] Uscinksi and Parent J. M. (2014). Uscinski, J.E. and Parent J.M. (2014), *American Conspiracy Theories*. Oxford University Press.

[CR105] van der Wal, R. C., Sutton, R. M., Lange, J., & Braga, J. P. N. (2018). Suspicious binds: Conspiracy thinking and tenuous perceptions of causal connections between co-occurring and spuriously correlated events. *European Journal of Social Psychology,**48*, 970–989. 10.1002/ejsp.2507 30555189 10.1002/ejsp.2507 PMC6282862

[CR204] Van Leeuwen, N. (2007). The Product of Self-Deception. *Erkenntnis, 67*, 419–437.

[CR106] van Prooijen, J.-W., & van Lange, P. A. M. (2014). The social dimension of belief in conspiracy theories. In J.-W. van Prooijen & P. A. M. van Lange (Eds.), *Power, Politics, and Paranoia: Why People are Suspicious of Their Leaders* (pp. 237–253) Cambridge University Press.

[CR107] van Prooijen, J.-W., & Acker, M. (2015). The influence of control on belief in conspiracy theories: Conceptual and applied extensions. *Applied Cognitive Psychology,**29*, 753–761.

[CR108] Van Prooijen, J.-W. (2019). Belief in conspiracy theories. Gullibility or Rational Skepticism?. In Joseph Forgas and Roy Baumeister (Eds.), *The Social Psychology of Gullibility. Fake News, Conspiracy Theories, and Irrational Beliefs* (pp. 319–332) Routledge

[CR109] Wakefield, J. C. (1992a). Disorder as Harmful Dysfunction: A conceptual critique of *DSM-III-R*’s definition of mental disorder. *Psychological Review,**99*(2), 232–247. 1594724 10.1037/0033-295x.99.2.232

[CR110] Wakefield, J. C. (1992b). The concept of mental disorder: On the boundary between biological facts and social values. *American Pyschologist,**47*(3), 373–388. 10.1037//0003-066x.47.3.373 1562108

[CR111] Wood, M. J., & Douglas, K. M. (2018). Conspiracy theory psychology: individual differences, worldviews, and states of mindindividual differences, worldviews, and states of mind. In J. E. Uscinski (ed.) *Conspiracy Theories and the People who Believe them* (pp. 245–256) Oxford University Press.

[CR112] Williams, D. (2021). Socially adaptive belief. *Mind & Language,**36*, 333–354.

[CR113] Young, A. W., Robertson, I. H., Hellawell, D. J., de Pauw, K. W., & Pentland, B. (1992). Cotard delusion after brain injury. *Psychological Medicine,**22*, 799–804. 1410102 10.1017/s003329170003823x

